# Solid-Binding Peptide-Guided Spatially Directed Immobilization of Kinetically Matched Enzyme Cascades in Membrane Nanoreactors

**DOI:** 10.1021/acsomega.1c03774

**Published:** 2021-10-04

**Authors:** Deniz T. Yucesoy, Susrut Akkineni, Candan Tamerler, Bruce J. Hinds, Mehmet Sarikaya

**Affiliations:** † Department of Materials Science and Engineering, University of Washington, Seattle, Washington 98195, United States; ‡ Department of Bioengineering, Izmir Institute of Technology, Urla, Izmir 35430, Turkey; § Department of Mechanical Engineering, Institute for Bioengineering Research, University of Kansas, Lawrence, Kansas 66045, United States

## Abstract

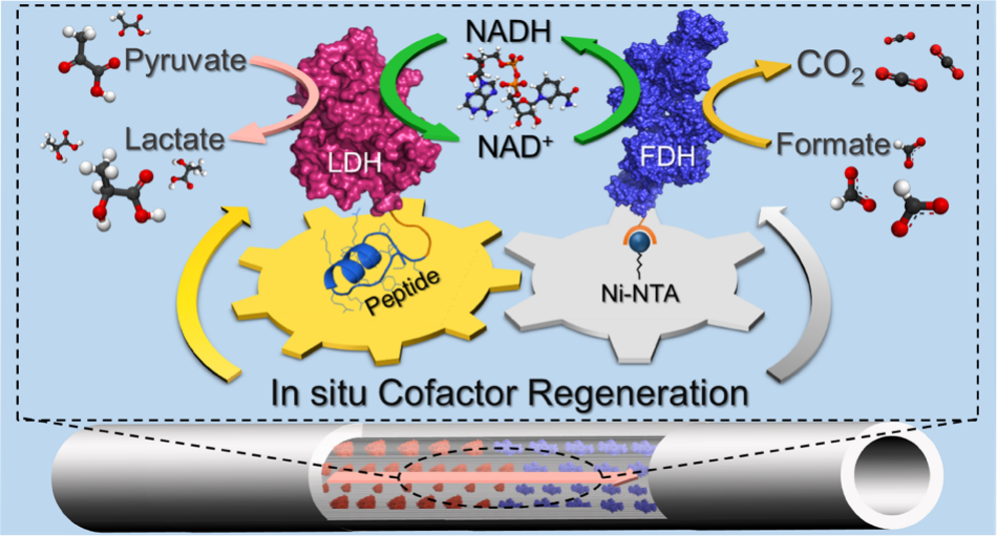

Biocatalysis is a useful strategy for sustainable green synthesis of fine chemicals due to its high catalytic rate, reaction specificity, and operation under ambient conditions. Addressable immobilization of enzymes onto solid supports for one-pot multistep biocatalysis, however, remains a major challenge. In natural pathways, enzymes are spatially coupled to prevent side reactions, eradicate inhibitory products, and channel metabolites sequentially from one enzyme to another. Construction of a modular immobilization platform enabling spatially directed assembly of multiple biocatalysts would, therefore, not only allow the development of high-efficiency bioreactors but also provide novel synthetic routes for chemical synthesis. In this study, we developed a modular cascade flow reactor using a generalizable solid-binding peptide-directed immobilization strategy that allows selective immobilization of fusion enzymes on anodic aluminum oxide (AAO) monoliths with high positional precision. Here, the lactate dehydrogenase and formate dehydrogenase enzymes were fused with substrate-specific peptides to facilitate their self-immobilization through the membrane channels in cascade geometry. Using this cascade model, two-step biocatalytic production of l-lactate is demonstrated with concomitant regeneration of soluble nicotinamide adenine dinucleotide (NADH). Both fusion enzymes retained their catalytic activity upon immobilization, suggesting their optimal display on the support surface. The 85% cascading reaction efficiency was achieved at a flow rate that kinetically matches the residence time of the slowest enzyme. In addition, 84% of initial catalytic activity was preserved after 10 days of continuous operation at room temperature. The peptide-directed modular approach described herein is a highly effective strategy to control surface orientation, spatial localization, and loading of multiple enzymes on solid supports. The implications of this work provide insight for the single-step construction of high-power cascadic devices by enabling co-expression, purification, and immobilization of a variety of engineered fusion enzymes on patterned surfaces.

## Introduction

Enzymatic pathways that perform multistep reactions in biological organisms are the key processes that enable life.^1−4^ Biomimetic reconstruction of these metabolic pathways by incorporating multiple enzymes and relevant cofactors in a confined environment is a highly valuable strategy for sustainable green synthesis of fine chemicals e.g., pharmaceuticals, biofuels, and consumer products, as well as developing efficient biomolecular devices.^5−9^ The presence of multiple enzymatic components, however, brings unique challenges, such as (i) controlling the spatial distribution of enzymes on the support surface; (ii) enabling efficient transport of reactive intermediates from one enzyme to another; and (iii) kinetically matching enzymes with different turn-over rates.^10−12^

An anodic aluminum oxide (AAO) membrane is a suitable platform to reconstruct enzyme assemblies due to the dominance of convective flow through their highly oriented monolithic channels, which facilitate efficient transport of reactive intermediates sequentially from one enzyme site to another.^13−16^ Using the well-established anodization processes, the size of the monolithic channels can be modified to control enzyme load and the flow behavior.^17−19^ Numerous efforts have been devoted to construct multienzyme assemblies on AAO using a variety of approaches.^20−23^ One major shortcoming of these approaches, however, is the utilization of the same coupling chemistry for the entire system, which limits the immobilization of the cascading components in the correct sequence.^20,22,24,25^ With their exquisite material recognition and self-assembly properties, solid-binding peptides are appealing engineering tools, which can be utilized as molecular linkers to selectively immobilize biomolecules, e.g., enzymes, on a variety of solid surfaces.^26−29^ In particular, by constructing AAO membranes functionalized with diverse materials, mixtures of fusion enzymes can be self-directed to immobilize on a desired target material layer, in a single step with high positional precision.

Solid-binding peptides are used as the fundamental molecular building blocks to facilitate the seamless integration of inorganic solid materials with biological molecules toward the development of hybrid devices and systems.^26,30−34^ These peptides are a short sequence of amino acids that were selected with affinity to inorganic solids using directed evolution techniques, such as phage display and cell surface display.^26,31^ The specificity of the solid-binding peptide for a given solid, e.g., gold, silica, or graphite, stems from the physicochemical effects at the interface that involve a combination of weak interactions, including van der Waals, hydrogen bonding, and coulombic forces, as well as surface diffusion and structural changes toward establishing a stable conformation with multiple contact points creating a unique footprint on the material surface.^30,33,35,36^

The authors and other groups have identified multitudes of solid-binding peptides that are specific to metals, ceramics, and minerals, e.g., Au, Ag, Pt, TiO_2_, SiO_2_, hydroxyapatite, and graphite.^35,37−43^ Their utility as anchoring moieties facilitating immobilization of functional proteins onto solid surfaces has been demonstrated at large length scales.^29,32,44−46^ With their exclusive specificity and binding properties, solid-binding peptides offer a superior alternative to traditional surface functionalization and activation approaches that utilize nonspecific physical adsorption or covalent bonding.^11,32,47,48^ The ease of genetic insertion of these short sequences to a permissive site or to the C- or N-terminus of proteins makes them highly useful heterofunctional molecular constructs.^11,47,49,50^ By providing addressable self-organization when combined with other functional biomolecules, e.g., DNA, RNA, enzymes, etc., in chimera, these peptides, therefore, are particularly well suited for immobilization of bioactive molecules providing much greater control over their binding, assembly, and orientation control on solid surfaces.^34,51,52^

**Figure 1:**
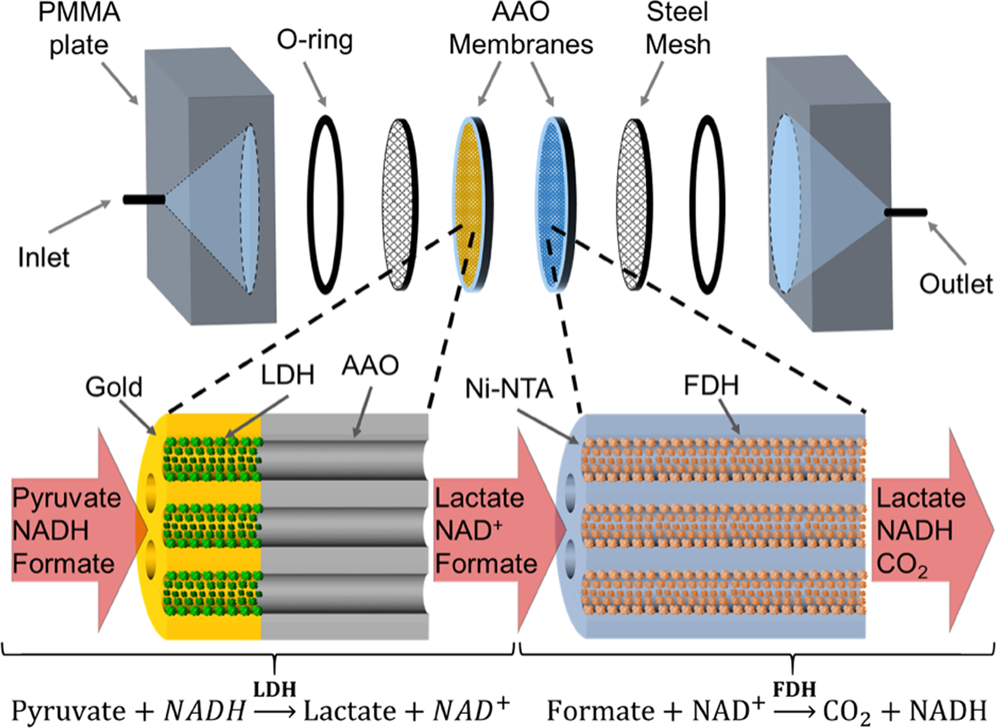
Schematic representation of the enzymatic flow bioreactor design. Gold- and Ni-NTA-functionalized AAO membranes are stacked together to sequentially immobilize genetically engineered fusion enzymes, *c*AuBP2-LDH and His-FDH, respectively.

The lactate dehydrogenase (LDH) and formate dehydrogenase (FDH) enzyme couple is a well-characterized cascade model.^53−55^ When utilized together, these enzymes catalyze a two-step biochemical reaction where in the first step LDH breaks down the pyruvate, the final product of glycolysis, into chiral lactate, as shown in Figure 1.^49,55^ This reaction is mediated by the oxidation of the nicotinamide adenine dinucleotide (NADH) cofactor into NAD^+^, acting as a hydrogen source necessary for lactate synthesis. In large-scale applications, especially in industrial settings, recycling of this relatively expensive NADH is crucial to develop cost-efficient synthesis processes.^56,57^ Regeneration of the nicotinamide cofactor (NAD^+^ to NADH) is facilitated by the second reaction catalyzed by the FDH enzyme, which breaks down the formate into carbon dioxide while reducing the NAD^+^ (produced by LDH) into NADH.^11,55−57^ Here, obtaining high yields of NADH regeneration depends on the positional precision of each enzyme on the solid support, where the LDH is assembled at the upstream of FDH. The LDH and FDH bienzyme system, therefore, offers a suitable model to test the efficacy of substrate-specific peptide tags in addressable self-immobilization of cascading enzymes on AAO membranes.

The aim of this study is to develop a robust flow bioreactor to enzymatically regenerate NADH by sequentially immobilizing the engineered LDH and FDH enzymes that can self-organize on gold- and the Ni-NTA-activated AAO surface. This goal has been achieved by genetically conjugating solid-specific peptide tags, specifically, the gold-binding peptide (*c*AuBP2)^41,44^ and conventional poly-histidine peptide (His),^58^ with the LDH and FDH enzymes, respectively. The efficacy of the peptide-guided immobilization approach was demonstrated by the positional precision of enzymes that allowed tunable stoichiometric arrangements, kinetic pairing, and increased catalytic yield of the flow bioreactor. Incorporating solid material specificity into biocatalysts via peptides could allow the construction of highly potent future hybrid nanodevices and high-power cascadic reactors by enabling co-expression, purification, and immobilization of a variety of fusion enzymes in a single step.

## Results and Discussion

The design of the enzymatic flow reactor is schematically described in Figure 1, where the engineered LDH and FDH fusion enzymes are self-immobilized by the genetically conjugated peptide tags to sequentially catalyze two-step regeneration of the NADH cofactor under continuous flow.

Below, the design and production of fusion protein constructs, kinetic characterization of the immobilized enzymes, and the modular configuration of the bienzymatic flow bioreactor and its catalytic performance are demonstrated. The high catalytic activity obtained under continuous operation indicates that the peptide-directed immobilization is a highly effective strategy to control surface orientation, spatial localization, and loading as well as the kinetic pairing of multiple enzymes on the same solid support.

### Design and Production of Fusion Enzymes

**Figure 2:**
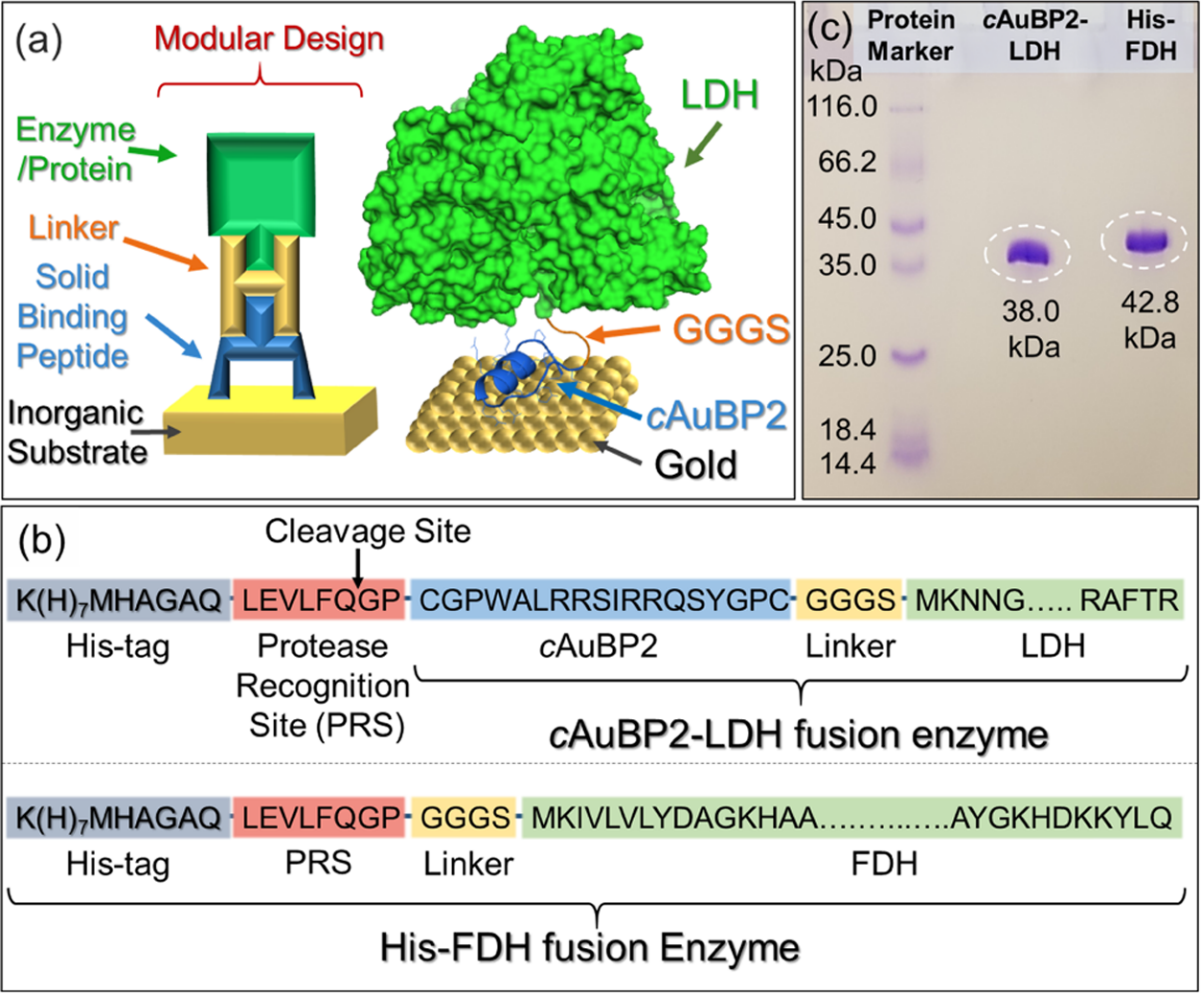
(a) Conceptual schematics of the modular fusion enzyme design strategy. (b) Sequence map of engineered His-*c*AuBP2-LDH and His-FDH fusion proteins with His-tag, PreScission Protease recognition, linker and *c*AuBP2 domains. (c) SDS-PAGE gel image of *c*AuBP2-LDH and His-FDH proteins after purification; the left lane shows the protein weight marker with corresponding molecular masses, while *c*AuBP2-LDH and His-FDH are shown in the middle and right lanes (dotted circles), respectively.

The genetic fusion of *c*AuBP2- and His-tags to LDH and FDH enzymes has been reported previously.^11,49^ Both fusion enzymes were designed to have the His domain on their N-terminal ends to facilitate the purification process (Figure 2a,b).

In the case of the *c*AuBP2-LDH fusion enzyme, an 18 amino acid long, combinatorially selected gold-binding peptide (*c*AuBP2) was inserted between His-tag and the N-terminus of the LDH.^11,49^ A site-specific protease recognition sequence was utilized to facilitate the removal of His-tag via specific proteases following the purification process. A flexible, <1 nm long spacer sequence, glycine–glycine–glycine–serine (GGGS), was integrated between the peptide tag and the enzyme to ensure that both domains are freely exposed to the environment without any restriction on their distinct functionalities (Figure 2b). Both engineered fusion enzymes, *c*AuBP2-LDH and His-FDH, were expressed in the *Escherichia coli* DH5α-T1 strain and purified using Ni-NTA matrices (Qiagen) under native conditions (see the Materials and Methods). The poly-histidine tag in His-*c*AuBP2-LDH was removed by the PreScission Protease (GE Healthcare) cleavage to eliminate nonspecific interactions between the poly-histidine tag and the gold surface. Upon cleavage and purification, both enzymes were exchanged into 5 × 10^–2^ M Tris Buffer, pH 7.5, and kept at 4 °C until use. The purity of fusion proteins was assessed by sodium dodecyl sulfate polyacrylamide gel electrophoresis (SDS-PAGE) and the molecular weights were confirmed as 38 and 42.8 kDa for *c*AuBP2-LDH and His-FDH, respectively (Figure 2c).

### Binding Kinetics and Catalytic Activities of Fusion Enzymes

The retained catalytic activities of each fusion enzyme following the peptide tag insertion were quantitatively analyzed by monitoring the oxidation of NADH (LDH catalyzed) or reduction of NAD (FDH catalyzed) at 340 nm. The catalytic turn-over rates were found to be 66.8 ± 3.7 and 0.59 ± 0.02 s^–1^ for *c*AuBP2-LDH and His-FDH, respectively.

**Figure 3:**
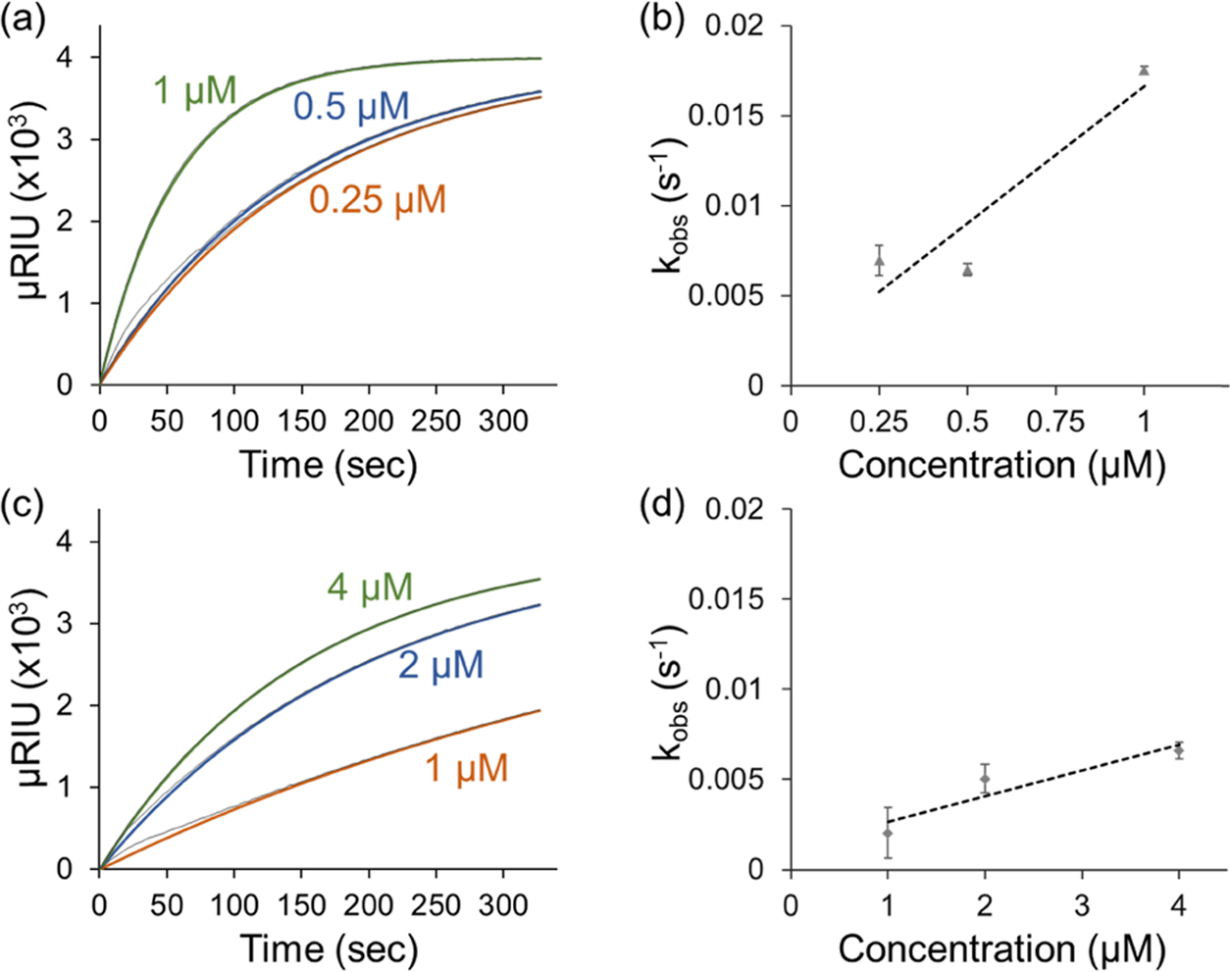
(a) Adsorption isotherms for the *c*AuBP2-LDH fusion enzyme obtained at 0.25, 0.5, and 1 μM concentrations. The dark gray lines in the background represent experimental data points, and the solid blue, green, and orange lines are 1:1 Langmuir model fits. (b) Observed rate coefficients (*k*_obs_) plotted as a function of the enzyme concentration. (c) Adsorption isotherms and (d) observed rate coefficients (*k*_obs_) for wild-type LDH obtained at 1-, 2-, and 4-μM concentrations. Linear regression was used to calculate the adsorption (*k*_a_) and desorption (*k*_d_) rates.

The specific gold-binding affinity of the *c*AuBP2-LDH fusion enzyme was quantitatively determined using surface plasmon resonance (SPR) spectroscopy (Figure 3). Adsorption spectra recorded at several concentrations were fitted using the 1:1 Langmuir adsorption model.^39^ The resulting adsorption rate (*k*_a_), desorption rate (*k*_d_), equilibrium constant (*K*_eq_), and free-energy change of adsorption (Δ*G*) are provided in Table 1. While no significant reduction was observed in the catalytic activities of enzymes after the peptide tag insertion, the affinity of *c*AuBP2-LDH to gold was shown to increase by an order of magnitude compared to wild-type LDH.

**Table 1:** Adsorption Rate (*k*_a_), Desorption Rate (*k*_d_), Equilibrium Coefficient (*K*_eq_), and Adsorption Free Energy (Δ*G*_ads_) of Enzymes

enzyme	*k*_a_ × 10^–4^ (M^–1^ s^–1^)	*k*_d_ × 10^–4^ (s^–1^)	*K*_eq_ (M^–1^)	Δ*G*_ads_ (kcal mol^–1^)
*c*AuBP2-LDH	152.4 ± 18.2	14.2 ± 2.9	10.7 ± 6.2	–1.45 ± 0.37
LDH	14.9 ± 2.6	12.3 ± 0.8	1.20 ± 1.5	–0.12 ± 0.03

### Volumetric Flow Rate Identification

An optimal cascade reactor design should enable the complete conversion of reactants at each catalyst site. This ensures the operation of the reactor with high efficiency under continuous flow while preventing the accumulation of intermediates and unreacted species. The two orders of magnitude difference between the observed turn-over rates of the *c*AuBP2-LDH (66.8 ± 3.7 s^–1^) and His-FDH (0.59 ± 0.02 s^–1^), therefore, necessitates the kinetic matching of these fusion enzymes prior to construction of the cascade reactor. The optimal volumetric flow rate in bioreactor systems yielding to complete enzymatic conversion can be calculated as the product of the number (moles) of the immobilized enzyme and its turn-over rate per substrate concentration. Therefore, in situations where catalytic activities of enzymes dramatically differ, it is expected that increasing the active load of the slower enzyme could enable pairing with faster catalytic partners at a particular flow rate.^16^ Solid-binding peptide tags with material specificity offer the unique opportunity to tune the active load of each enzyme by simply adjusting the accessible area of the target material, in this case gold- and Ni-NTA-coated AAO surfaces. It is important to note that further fine-tuning of the residence time at a set system volume flow rate can be achieved with pore-size reduction by electroless plating that would decrease residence time but reduce enzyme surface area.

In our design, while the gold coating was restricted to the top surface and the first 20 nm of the pore walls, the entire surface of the second membrane including the pores was functionalized with Ni-NTA (Figure 1). Total enzyme load in each reactor bed (AAO membrane) was calculated by subtracting the amount of the unbound enzyme from the initial enzyme load (quantified via Bradford protein assay) and found as 2.2 × 10^–7^ g (∼8.6 × 10^11^ molecules) and 1.4 × 10^–6^ g (∼9.9 × 10^12^ molecules) for *c*AuBP2-LDH and His-FDH, respectively. These findings demonstrate that ∼12 times higher His-FDH immobilization was achieved by selectively increasing the active Ni-NTA-functionalized area on the second membrane. Based on these measured enzyme loads, as the starting point, the theoretical volumetric flow rate enabling the complete catalytic conversion was calculated as 2.8 × 10^–2^ and 2.9 × 10^–3^ mL min^–1^, for *c*AuBP2-LDH and His-FDH, respectively (see the Supporting Information, Figures S1 and S2).

In the next step, to experimentally identify optimum cascade reactor operation parameters, the biocatalytic efficacy of each enzyme under continuous flow was explored in separate reactor beds toward (i) the synthesis of lactate by *c*AuBP2-LDH and (ii) the breakdown of formate by His-FDH. Gold- and Ni-NTA-functionalized AAO membranes were encased within the custom-made poly(methyl methacrylate) (PMMA) flow cell (Figure S1) and enzyme immobilization was performed by feeding each reactor with 3 mL of the respective fusion enzyme (5 × 10^–6^ M) using a syringe pump.

**Figure 4:**
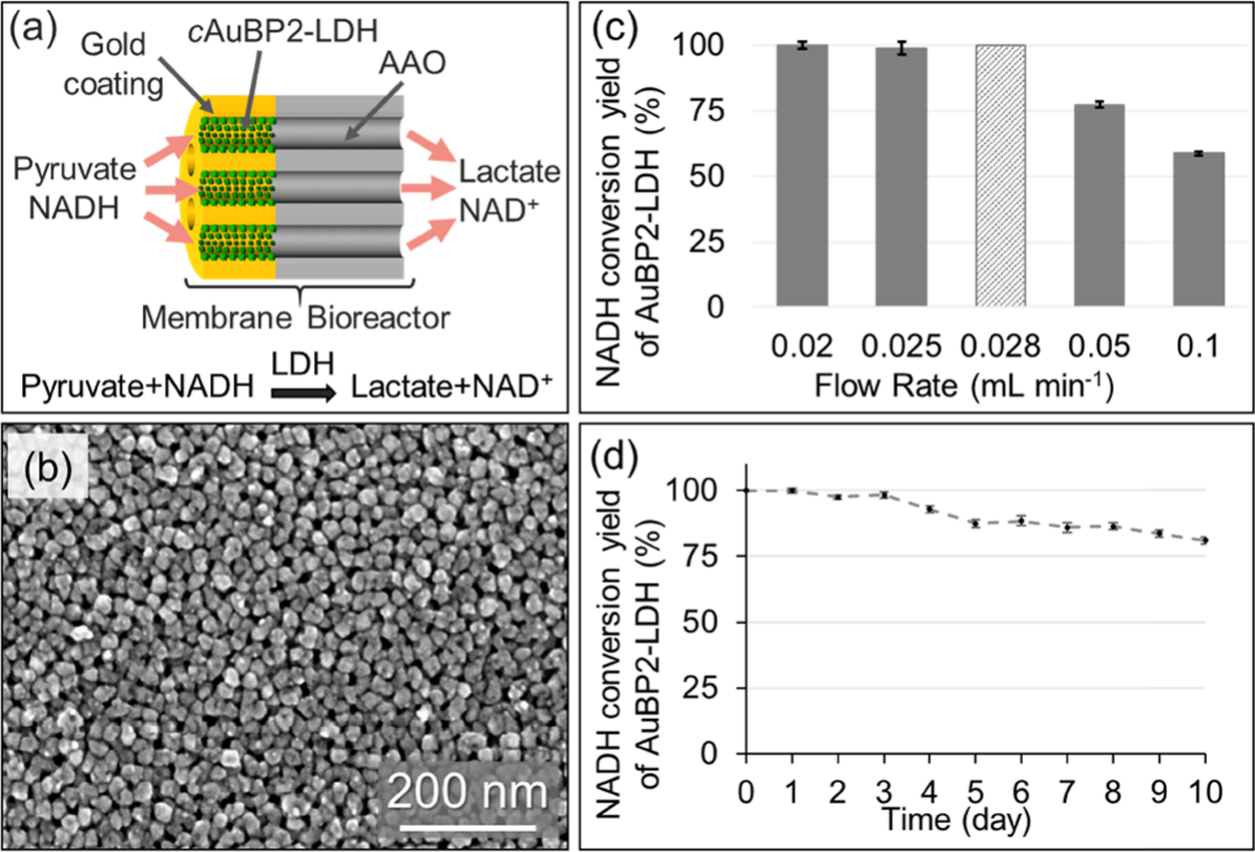
(a) Schematics of the *c*AuBP2-LDH flow bioreactor design. (b) Representative scanning electron microscopy image of the gold-functionalized AAO membrane. (c) Observed NADH conversion yield as a function of the flow rate of 0.02, 0.025, 0.05, and 0.1 mL min^–1^ in the *c*AuBP2-LDH flow bioreactor. The light gray (dashed) bar shows theoretical yield at the matched volumetric flow rate. (d) Operational stability of the *c*AuBP2-LDH flow bioreactor. Observed NADH conversion yield at the 0.02 mL min^–1^ flow rate as a function of time.

The first bioreactor bed constructed with the gold-functionalized AAO membrane and *c*AuBP2-LDH was then initialized by feeding the system with 2 × 10^–4^ M NADH and 2.5 × 10^–4^ M pyruvate (in 5 × 10^–2^ M Tris) reactant solution at 0.1, 0.05, 0.025, and 0.02 mL min^–1^ flow rates (Figure 4a,b).

Under continuous flow, *c*AuBP2-LDH catalyzes the breakdown of pyruvate with concomitant oxidation of NADH to NAD^+^ according to
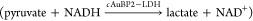
1The conversion products were collected in multiple fractions (∼150 μL) for 2 h at each flow rate and quenched immediately by mixing with the urea (8 M) solution. Each fraction was analyzed at 340 nm (NADH absorption wavelength) using a spectrophotometer and unreacted NADH concentrations were calculated by using a linear calibration curve with standard solutions within the range of 0–10^–3^ M (see the Supporting Information, Figure S2). As shown in Figure 4c, at the 0.1 mL min^–1^ inward flow rate, only 58.8 ± 0.8% of the NADH was oxidized into NAD^+^. By slowing down the flow rate, the conversion efficacy of the bioreactor increased to its near ideal rate of 99.9 ± 1.2% at 0.02 mL min^–1^.

**Figure 5:**
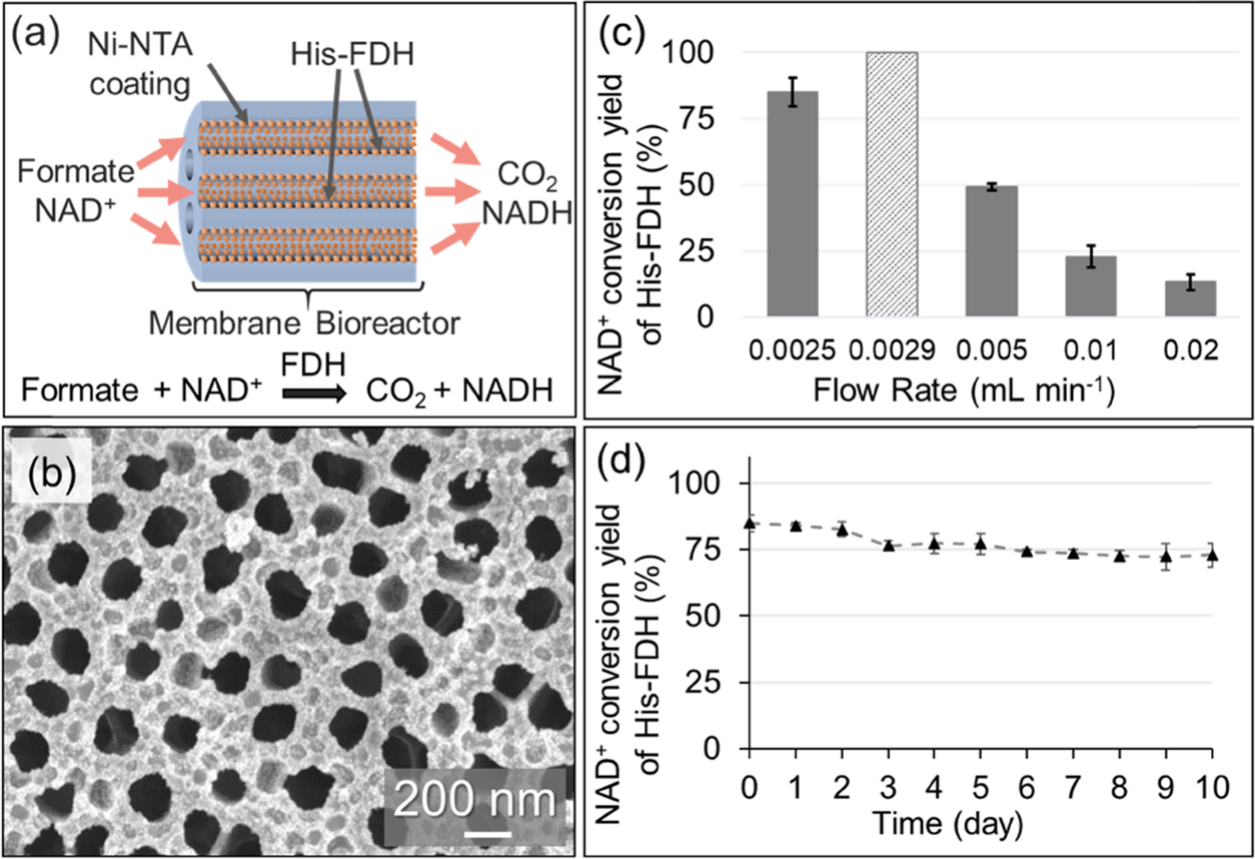
(a) Schematics of the His-FDH flow bioreactor design. (b) Representative scanning electron microscopy image of the Ni-NTA-coated AAO membrane. (c) Observed NAD^+^ conversion yield as a function of the flow rate of 0.0025, 0.005, 0.01, and 0.02 mL min^–1^ in the His-FDH flow reactor. The light gray (dashed) bar shows theoretical yield at the matched volumetric flow rate. (d) Operational stability of the His-FDH flow bioreactor. Observed NAD^+^ conversion yield at the 0.0025 mL min^–1^ flow rate as a function of time.

The second bioreactor was constructed with the Ni-NTA-functionalized AAO membrane and His-FDH enzyme (Figure 5a,b). In this reaction, His-FDH catalyzes the breakdown of formate with concomitant reduction of NAD^+^ to NADH according to
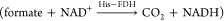
2The bioreactor was fed with 2 × 10^–4^ M NAD^+^ and 2.5 × 10^–4^ M formate (in 5 × 10^–2^ M Tris) solution at different flow rates and formation of NADH was monitored. The conversion efficacy of the His-FDH bioreactor was found to be 13.2 ± 3.1% at 0.02 mL min^–1^ (Figure 5c). By slowing down the flow rate to 0.0025 mL min^–1^, 85.1 ± 5.3% NAD^+^ reduction was obtained. It is noted that the difference between the turn-over rates of *c*AuBP2-LDH and His-FDH was originally ∼112-fold (Table 1). By increasing the (∼12 times) load of the slower enzyme in the flow bioreactor, the rate of the His-FDH-catalyzed reaction was successfully brought to an order of magnitude closer to the rate of the *c*AuBP2-LDH-catalyzed fast reaction, at the 0.02 mL min^–1^ flow rate. These findings also demonstrate that the modular configuration of the AAO bioreactor would bring these reactions to even much closer rates by simply using stacked layers of multiple Ni-NTA-activated AAO membranes to further increase the His-FDH load in the system. While an excellent agreement was obtained between the experimentally measured and theoretically calculated volumetric flow rates of the *c*AuBP2-LDH bioreactor (Figure 4d), in the case of His-FDH 20% deviation was observed (Figure 5d), presumably due to the restriction of active sites of the enzyme as a result of suboptimal surface orientation as well as supramolecular interaction of the enzymes with each other. It is crucial to note that each reactor before and after enzyme immobilization was confirmed to be free of nonspecific oxidation and reduction of the nicotinamide cofactor by the gold- and Ni-NTA-coated membranes as well as by the enzymes in the absence of pyruvate and formate.

The stabilities of the *c*AuBP2-LDH and His-FDH nanoreactors were tested during continuous operation at room temperature for 10 days at 0.020 and 0.0025 mL min^–1^ flow rates, respectively. The catalytic activities were seen to decrease gradually over time. After 10 days of continuous operation at room temperature, 84.7 and 80.8% of the initial catalytic activities of His-FDH (Figure 5d) and *c*AuBP2-LDH (Figure 4d) were preserved, respectively.

### Construction of the Bienzymatic Cascade Flow Reactor

Following the optimization of volumetric flow rates for each fusion enzyme and confirming their operational stability under the optimized conditions, in the next step, the cascade reactor involving both enzymes in the single flow cell was constructed. In the two-step reaction, the *c*AuBP2-LDH and His-FDH fusion enzymes catalyze the synthesis of chiral lactate from pyruvate with *in situ* NADH regeneration according to eq 3 below.
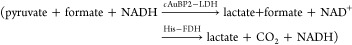
3The *c*AuBP2-LDH/His-FDH system is a suitable cascade model to demonstrate the efficacy of orthogonal peptide tags as fusion partners to enable the addressable immobilization of the enzymes across the AAO membrane channels. In this configuration (eq 3), misplacement of the fusion enzymes, e.g., immobilization of LDH to the downstream of FDH, would lead to reconversion of NADH/NAD^+^ through the length of the AAO channels, which, in turn, decreases the *in situ* NADH regeneration efficacy of the flow reactor. The dual-membrane cascade reactor was constructed by assembling gold (top)- and Ni-NTA (bottom)-functionalized membranes together within a PMMA flow cell. Enzyme immobilization was then performed by pumping 3 mL’s of *c*AuBP2-LDH and His-FDH fusion enzymes into the flow cell.

**Figure 6:**
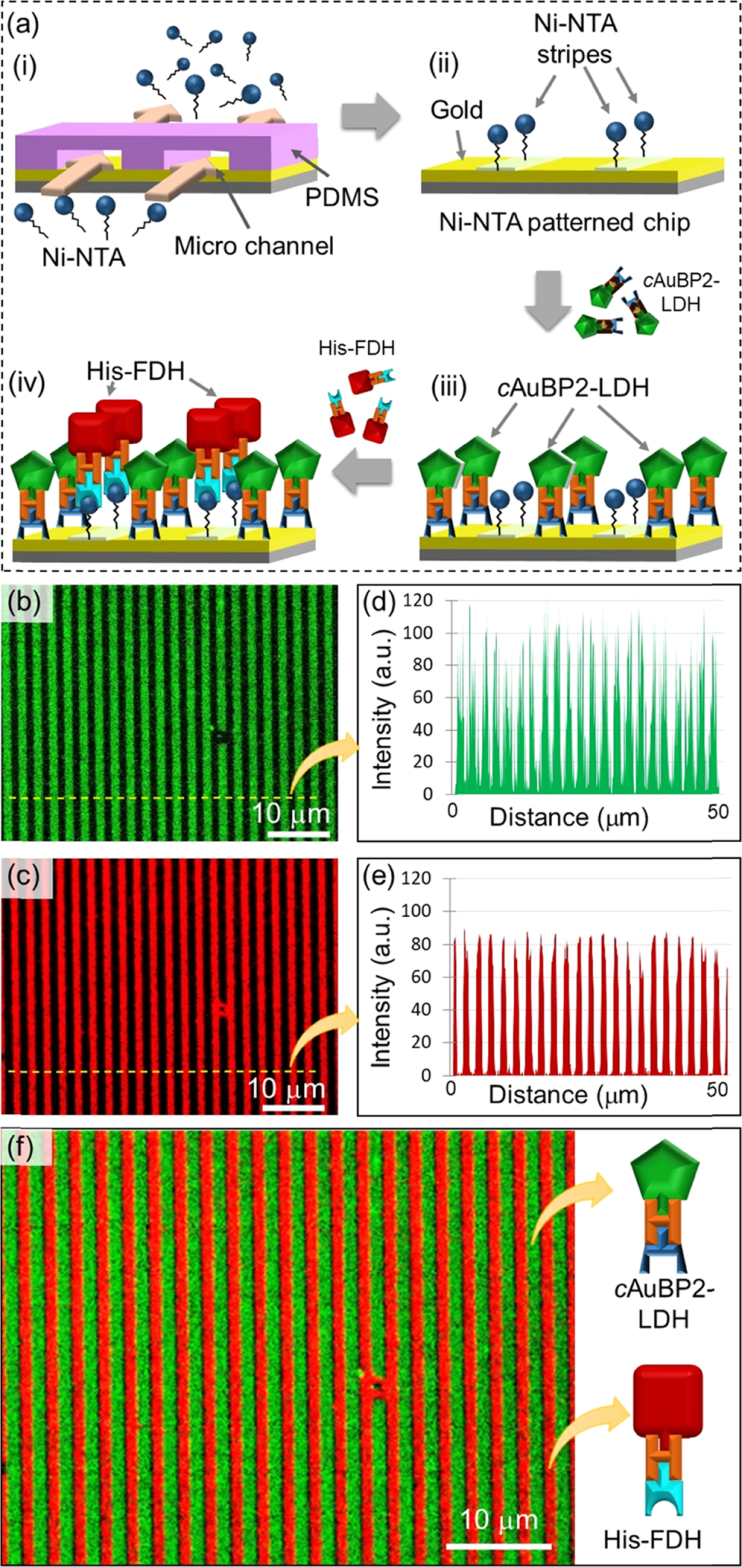
(a) Schematics of the peptide-directed immobilization of fluorescently labeled His-FDH and *c*AuBP2-LDH fusion enzymes on the Ni-NTA-patterned gold surface. (b) Fluorescence microscopy image of *c*AuBP2-LDH micropatterns immobilized on the Ni-NTA-patterned gold surface. The image was recorded using a 5-FAM filter and green stripes show *c*AuBP2-LDH. (c) Fluorescence microscopy image of His-FDH micropatterns immobilized on Ni-NTA regions on the gold surface. The image was recorded using a QD605 filter and red stripes show His-FDH. (d, e) Respective line scan plots of fluorescence images generated using ImageJ software. (f) Digital overlay of the images (b, c).

Gold surfaces are particularly prone to nonspecific interactions by a wide variety of proteins often requiring inert coatings such as poly(ethylene glycol) (PEG).^59^ Thus, one of the design challenges in the bienzyme system is to immobilize *c*AuBP2-LDH and His-FDH onto their respective membranes with high precision while pumping both enzymes into the flow cell. Although peptide tags provide selective binding, the nonspecific adsorption of His-FDH on gold-coated areas is possible due to the accessible cysteine and histidine residues of the FDH enzyme. To circumvent this challenge, we hypothesized that by performing *c*AuBP2-LDH loading initially, the accessible gold area in the flow bioreactor could be saturated and, thereby, the nonspecific adsorption of His-FDH on the gold-coated membrane would be prevented. This hypothesis was tested using a Ni-NTA micropatterned gold substrate using fluorescently labeled *c*AuBP2-LDH and His-FDH enzymes (Figure 6a).

The Ni-NTA-micropatterned gold chip was initially immersed into a 5-carboxyfluorescein (5-FAM)-conjugated *c*AuBP2-LDH solution for 10 min and then the excess enzyme was rinsed off with 2 × 10^–2^ M imidazole (in 5 × 10^–2^ M Tris, pH 7.5) solution. Next, the gold chip was placed into Qdot-labeled His-FDH solution. After washing off the excess His-FDH, the gold substrate was air-dried and imaged with fluorescent microscopy using 5-FAM (excitation and emission wavelengths; 492–518 nm) and Qdot (excitation and emission wavelengths; 300–603 nm) filters.

As shown in Figure 6b,c, a step-by-step enzyme immobilization approach resulted in a high-fidelity assembly of *c*AuBP2-LDH and His-FDH fusion enzymes, appearing as sharp alternating red and green strips. It should be noted that both images in Figure 6b,c were recorded from the same area of the sample. While the green bands (pseudocolored) represent 5-FAM-conjugated *c*AuBP2-LDH (Figure 6b) immobilized on the gold surface, the red regions in between display Qdot-labeled His-FDH (Figure 6c) enzymes assembled on previously unoccupied Ni-NTA-coated surfaces. The distances between the red and green bands and their respective signal intensities were further quantified using line profiling (Figure 6d,e). The average distance between the peaks was calculated using full-width half-maximum analysis and found as 1.08 ± 0.06 μm. This distance is consistent with the line width of the PDMS stamp (∼1.2 μm) that is utilized to fabricate the Ni-NTA micropatterns on the gold substrate. To demonstrate the fidelity of alternating patterns even more clearly, the fluorescent images were digitally overlaid (Figure 6f) using ImageJ software (NIH, Bethesda, MD) showing the perfect fit between the two patterned strips. These results demonstrate that using the step-by-step immobilization approach, fusion enzymes harboring different peptide tags can be selectively immobilized onto their respective surfaces.

After confirming the efficacy of the step-by-step immobilization procedure, in the next step, a dual-bed cascade reactor was constructed by pumping the *c*AuBP2-LDH into the flow cell that is followed by His-FDH injection. The 0.0025 mL min^–1^ was chosen as an operational flow rate for the bienzyme flow reactor at which *c*AuBP2-LDH catalyzes complete oxidation of NADH while His-FDH regenerates 85.1 ± 5.3% of NAD^+^ back to NADH (Figure 5c). The system was fed with 2 × 10^–4^ M NADH, 2.5 × 10^–4^ M formate, and 2.5 × 10^–4^ M pyruvate (in 5 × 10^–2^ M Tris) reactant solution and the efficacy of the cascade reaction was measured by monitoring the NADH concentration in the outlet. It is noted that, before each run, a complete enzymatic oxidation of NADH via *c*AuBP2-LDH was confirmed by feeding the system with 2 × 10^–4^ M NADH and 2.5 × 10^–4^ M pyruvate (in 5 × 10^–2^ M Tris) reactant solution (containing no formate) at 0.0025 mL min^–1^.

**Figure 7:**
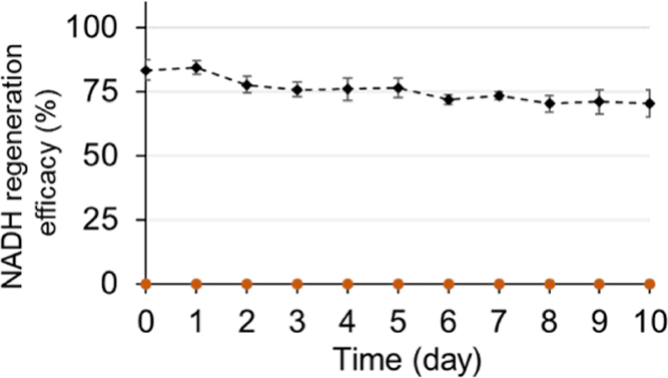
Observed NADH regeneration yield of *c*AuBP2-LDH and His-FDH two-enzyme flow bioreactors at the 0.02 mL min^–1^ flow rate as a function of time (dark gray diamonds). Formate ion production (% of feed concentration) as a function of time (orange dots) was less than the detection limit.

As shown in Figure 7, in the absence of formate, no NADH was detected in any of the fractions collected from the reactor output (detection limit ∼2 × 10^–6^ M). This result indicates that the enzymatic oxidation of NADH by *c*AuBP2-LDH activity had been complete. In the presence of formate, however, 83.3 ± 3.8% of initial NADH concentration was obtained in the output, which clearly demonstrates that 100% of the NADH was initially oxidized into NAD^+^ by the activity of *c*AuBP2-LDH and then 83.3 ± 3.8% of it was successfully regenerated by the second reaction catalyzed by the His-FDH fusion enzyme.

The regeneration yield achieved here was quite close to the efficacy of the His-FDH single-enzyme bioreactor (85.1 ± 5.3% conversion yield) that was constructed initially (Figure 5c). As indicated previously, any mislocalized enzymes within the pore channels can lead to reconversion of NADH/NAD^+^, which in turn decreases the regeneration efficacy of the flow reactor. Thus, obtaining similar conversion rates in both His-FDH single-enzyme and *c*AuBP2-LDH/His-FDH dual-enzyme configurations suggests that the accurate localizations of enzymes on their respective surfaces were achieved in the dual-membrane cascade reactor design.

Next, using the same operational conditions, the stability of the system under continuous flow was tested. In a given bioreactor design, the long-term stability of active molecules, e.g., enzymes, is essential for its implementation on an industrial scale.^3,4^ The NADH regeneration efficacy of the dual-membrane reactor was monitored under continuous operation at room temperature for 10 days at the 0.0025 mL min^–1^ flow rate. As shown in Figure 7, the amount of regenerated NADH in the outlet solution was seen to decrease gradually over time. This could be attributed to partial inactivation of the enzymes under continuous operation at room temperature due to their limited thermal stability as well as their leaching from the reactor bed. During the operation of the reactor, the system was fed with the solution containing only the substrate and cofactor molecules. As the enzyme introduction into the reactor was discontinued, dynamic equilibrium created at the membrane–solvent interface shifts toward dissociation due to the nature of the electrostatic interactions, e.g., electrostatic forces, van der Waals forces, hydrogen bonding, etc., formed between the peptide tag and the activated membrane surface. Although being highly superficial, this results in slow leaching of the immobilized enzymes from the reactor bed leading to a gradual decrease in reactor efficacy.^30,33,35,36^ However, even after 10 days of continuous operation at room temperature, 84.4% of the initial catalytic performance was maintained, which is significantly higher than the stabilities of LDH and FDH cascades that are reported as 55–70%, previously.^60,61^

## Conclusions

We developed a modular cascade flow reactor using a generalizable strategy based on the peptide-directed immobilization approach that enables selective self-immobilization of fusion enzymes on AAO monoliths with high positional precision. Material-specific fusion enzymes were constructed by genetic insertion of the combinatorially selected gold-binding peptide (*c*AuBP2) and a widely used poly-histidine (His-tag) tag into the N-terminus of the LDH and FDH enzymes, respectively. While retaining their catalytic activities, both fusion enzymes exerted a high degree of selective binding on Au and Ni-NTA surfaces, respectively, suggesting that substrate specificity is imparted by the solid-specific peptide tags. The structurally flexible spacer sequence (GGGS) integrated between the enzyme and peptide tags enabled the orthogonal immobilization of the fusion enzymes without restricting their catalytic activity on the substrate surface, the major limitation of covalent immobilization approaches.^20,22,24^

Utilizing the LDH/FDH cascade model, fusion enzymes were self-immobilized onto the gold- and Ni-NTA-functionalized AAO membranes in a cascade geometry to enable the two-step production of l-lactate with concomitant *in situ* NADH regeneration. Here, the material specificity of the peptide tags not only facilitated the cascading arrangement of the fusion enzymes along the AAO channels but also allowed the precise control of enzyme loading, which is crucial for the kinetic pairing of multiple enzymes with different catalytic efficiencies. Precisely formed enzyme patterns on the Ni-NTA-coated gold surface (Figure 6) as well as high NADH regeneration efficacy (Figure 7) demonstrate that the peptide-directed immobilization approach addresses the kinetic coupling problem in multienzymatic systems often curtailed by random and nonspecific immobilization strategies. Furthermore, the dominance of the convective flow along the AAO monoliths appeared to have mitigated the accumulation of unwanted side reactions, e.g., NADH reconversion, thereby resulting in significantly enhanced catalytic conversion output.

In short, the peptide-directed immobilization procedure described in this work offers a highly effective strategy to control surface orientation, spatial localization, and individual loading of cascading enzymes onto a variety of solid supports. Empowered by the large repertoire of solid-specific peptide tags specific to different materials, e.g., Au, Pt, Ag, Al_2_O_3_, SiO_2_, graphite, etc., the modular reactor design outlined herein can be further extended to build complex catalytic cascades, such as the complete glycolysis pathway, as well as the construction of novel synthetic routes for the biocatalytic synthesis of fine chemicals and therapeutics. Such complex multienzyme systems, with the directed sequential flow and tuned residence time, can be efficiently fabricated in a single-step procedure by capturing the co-expressed fusion enzymes directly from bacterial lysates without a need for further isolation, purification, and immobilization processes toward highly potent industrial implementations.

## Materials and Methods

### Materials

Sixty micrometers thick and 1.82 cm diameter AAO membranes with 20–200 nm nominal pore sizes were purchased from Whatman (Maidstone, U.K.). Gold(I) thiosulfate was obtained from Alfa Aesar (Ward Hill, MA). All other chemicals and reagents were purchased from Sigma-Aldrich (Milwaukee, WI) and used as received unless otherwise noted.

### Membrane Surface Functionalization

The AAO membrane with 20–200 nm nominal pore sizes was activated with gold using a procedure reported previously.^16^ Briefly, a 5 nm thick gold (Au) seed layer was sputtered on the 20 nm pore side of the membrane with no intermediate wetting layer using a Cressington Coating System (Ted-Pella) with a calibrated quartz crystal thickness monitor at a background pressure of 0.02 mbar operating at 100 W. Next, electroless plating was performed in 50 mM phosphate buffer, pH 7.0, containing 1.6 mM sodium gold(I) thiosulfate and 2.68 × 10^–3^ M ascorbic acid for 65 min. The second membrane with 20–200 nm pore sizes was functionalized with Ni-NTA using a multistep platinum deposition procedure.^62^ First, 20 nm of the Au–Pd alloy was sputtered on both faces of the membrane. The membrane was rinsed with isopropyl alcohol to pre-wet, followed by a quick wash with DI water, and then incubated in 0.1 M CuSO_4_ solution for 10 min. Next, using the Au–Pd-sputtered membrane faces as a working electrode, the reduction potential of the Cu surface plating layer was measured by cyclic voltammetry in a three-electrode electrochemical cell (Pt coil counter electrode and Ag/AgCl as the reference electrode) containing 5 × 10^–3^ M CuSO_4_ + 0.5 M H_2_SO_4_. Then, the deposition of the Cu surface plating layer through the 60 μm deep pore channels was ensured by applying the measured reduction potential for 10 min using the chronoamperometry method. Following the Cu surface plating layer deposition with a uniform thickness, the membrane was soaked in 1 mM PtCl_2_ solution for 30 min to ensure Pt displacement. Finally, the Ni-NTA monolayer was self-assembled on the Pt-coated membrane surface by immersing it in 2 × 10^–3^ M *N*-[*N*α,*N*α-bis(carboxymethyl)-l-lysine]-12-mercaptododecanamide (NTA) solution for 20 h followed by incubation in 0.1 M NiCl_2_ solution for 4 h at 25 °C. After functionalization, both membranes were rinsed with MilliQ water, dried with argon gas, and stored in a dry atmosphere. It is noted that the two-membrane system used as the bienzyme cascade support was constructed by bonding the polypropylene rings of the AAO membranes together to form a monolithic membrane without dead volume between membranes. Membranes were imaged before and after surface functionalization via an FEI Sirion XL30 SEM scanning electron microscope and the nominal pore size was determined using the ImageJ software. Prior to the construction of reactor beds, the maximum loading capacity of the Ni-NTA self-assembled monolayer was evaluated by the his-tagged green fluorescent binding protein (His-GFP) assay. Briefly, the Ni-NTA-functionalized membrane with the 20–200 nm pore size was soaked into 0.1 mL of 5 × 10^–5^ g mL^–1^ His-GFP (EMD Millipore) solution (in 1× Dulbecco’s phosphate-buffered saline (DPBS) buffer, pH: 7.4) and incubated for 15 min. Next, both faces of the membrane were rinsed with DPBS solution, and then, the membrane was soaked in 0.2 mL of 0.2 M imidazole solution (in 1× DPBS buffer, pH: 7.4) for 5 min to release the bound His_6_-GFP. The total fluorescence intensity of His-GFP in the imidazole solution was recorded using a fluorescence spectrophotometer (SpectraMax i3x, Molecular Devices) with a 395 nm excitation filter and a 509 nm emission filter. The amount of released His-GFP was quantified by plotting recorded absorbance values into a standard curve and fitted with a linear equation. The total His-GFP loading capacity on the Ni-NTA-functionalized membrane was 1.13 × 10^–6^ g.

### Peptide-Tagged Recombinant Fusion Enzyme Production

Bacterial expression and purification of fusion enzymes were done using a procedure described previously.^11,49^ Briefly, recombinant *E. coli* strains (DH5α-T1) harboring His-FDH and His-*c*AuBP2-LDH expression plasmid constructs were grown in the LB medium. The expression of both enzymes was induced by adding IPTG (isopropyl-β-d-thiogalactopyranoside) at OD_600_ of 0.5 to a final concentration of 5 × 10^–4^ M. The expressed protein was purified on Ni-NTA resin (Qiagen, Valencia, CA) under native conditions by following the manufacturer’s instructions. The purity of eluted enzymes was analyzed by sodium dodecyl sulfate polyacrylamide gel electrophoresis (SDS-PAGE) analysis. The hexa-histidine tag in the His-*c*AuBP2-LDH fusion enzyme was removed by the PreScission Protease (GE Healthcare) cleavage procedure described previously.^11,49^ Finally, both enzymes were exchanged to 5 × 10^–2^ M Tris buffer, pH 7.5, and kept at 4 °C until use. The enzyme concentration was determined by Bradford assay using bovine serum albumin (BSA) standards, at a wavelength of 595 nm.

### Binding Kinetics Characterization

The binding kinetics of the enzymes on gold was measured using surface plasmon resonance spectroscopy (Reichert Instruments). The baseline was established by feeding the flow cell with 5 × 10^–2^ M Tris Buffer, pH 7.5, until the refractive index change is stabilized. Wild-type LDH and *c*AuBP2-LDH enzymes obtained in 5 × 10^–2^ M Tris Buffer were then introduced at different concentrations and the change of the refractive index was recorded. The data from each enzyme concentration were fitted to a 1:1 Langmuir adsorption isotherm, and the adsorption rate (*k*_a_), desorption rate (*k*_d_), equilibrium coefficient (*K*_eq_), and Gibbs free energy for adsorption (Δ*G*_ads_) of enzymes were calculated, as described previously.^39^

### Flow Nanoreactor Assembly and Enzyme Immobilization

Flow nanoreactors were constructed by assembling surface-functionalized membranes into custom-made PMMA flow cells (Figure S1). Prior to any enzyme immobilization, flow nanoreactors were equilibrated 2 × 10^–2^ M imidazole (in 5 × 10^–2^ M Tris, pH 7.5) buffer solution with a constant flow of 0.1 mL min^–1^ for 30 min. In single-enzyme-containing flow reactors, immobilization was performed by feeding each flow cell with 3 mL of the respective fusion enzyme (5 × 10^–6^ M) using a syringe pump. The excess amount of the enzyme was removed by flowing through 5 mL of 2 × 10^–2^ M imidazole (in 5 × 10^–2^ M Tris, pH 7.5) buffer solution. Next, the system was equilibrated with 5 mL of 5 × 10^–2^ M Tris, pH 7.5, buffer solution at a flow rate of 0.1 mL min^–1^. In a bienzyme system, 3 mL of *c*AuBP2-LDH (5 × 10^–6^ M) was introduced to the pre-equilibrated flow cell and the excess amount was washed off with 2 × 10^–2^ M imidazole (in 5 × 10^–2^ M Tris, pH 7.5) buffer solution. Next, 3 mL of His-FDH (5 × 10^–6^ M) was introduced to the system via reserve flow from the outlet followed by the 5 mL of 2 × 10^–2^ M imidazole (in 5 × 10^–2^ M Tris, pH 7.5) buffer solution flow. Finally, the bienzyme flow reactor was re-equilibrated with 5 × 10^–2^ M Tris, pH 7.5, buffer solution. The amount of actual *c*AuBP2-LDH and His-FDH loading on gold- and Ni-NTA-functionalized AAO membranes, respectively, was quantified using Bradford assay. Briefly, during enzyme immobilization, the unbound *c*AuBP2-LDH and His-FDH were collected from the reactor outlet and quantified by Bradford assay using bovine serum albumin (BSA) standards, at a wavelength of 595 nm. The amount of the immobilized enzyme on each reactor was then determined by subtracting the collected amount from the initial enzyme load.

### Fluorescent Labeling of Fusion Enzymes

Using the 5-FAM protein labeling kit, 5-carboxyfluorescein (5-FAM) was conjugated with *c*AuBP2-LDH (Anaspec). Briefly, 5 × 10^–5^ M *c*AuBP2-LDH (in 10^–2^ M borate buffer, pH 7.4) was mixed with reaction buffer (component B) by vortexing. Then, 2 × 10^–5^ M 5-FAM solution (in dimethyl sulfoxide) was added onto enzyme solution with a 1:1 (v/v) ratio and incubated for 1 h at room temperature. Next, the reaction was quenched with 0.1 mL of glycine (0.1 M) solution for 1 h. The unreacted 5-FAM was removed by filtering the solution through a 30 kDa cutoff ultrafiltration centrifugal unit (Amicon). The Qdot-His-FDH conjugates were prepared by conjugating the amine residues of His-FDH with carboxyl-coated Qdot 605 (ThermoScientific) through the EDC/sulfo-NHS chemistry. Briefly, the Qdot stock solution was diluted in 10^–2^ M borate buffer (pH 7.4) to a final concentration of 1 × 10^–7^ M. Then, 0.5 mL of this solution was mixed with an equal volume of His-FDH (5 × 10^–5^ M) solution. Conjugation reaction was started by adding freshly prepared 0.1 mL of EDC/NHS solution (1 mg mL^–1^) and incubated for 2 h by continuous stirring at room temperature. The unreacted carboxyl groups were quenched with 0.1 mL of glycine (0.1 M) solution for 1 h. The unreacted Qdot was removed by purifying the solution through the Ni-NTA matrices. Finally, the unlabeled His_6_-FDH was removed by filtering the solution through a 100 kDa cutoff ultrafiltration centrifugal unit (Amicon). After labeling, both enzymes were exchanged into 5 × 10^–2^ M Tris, pH 7.5, buffer solution.

### Ni-NTA-Patterned Gold Chip Construction and Fluorescent Microscopy Analysis

The poly(dimethylsiloxane) (PDMS) stamps were prepared, as reported previously.^38^ To create Ni-NTA patterns, PDMS stamps were placed onto the gold-coated chip (Reichert Instruments) and 2 × 10^–3^ M *N*-[*N*α,*N*α-bis(carboxymethyl)-l-lysine]-12-mercaptododecanamide (NTA) solution was flowed through the microchannels for 24 h (∼1.2 μm line width). Next, the PDMS stamp was removed and the gold-coated chip was placed into 0.1 M NiCl_2_ solution and incubated for 4 h at room temperature. Finally, the patterned chip surface was washed with deionized water and dried. Following the protein immobilization, the chips were mounted on a Nikon Eclipse TE-2000U fluorescence microscope (Nikon, Japan) coupled with a Hamamatsu ORCA-ER CCD camera. Filter sets (5-FAM and QD605) are used for *c*AuBP2-LDH and His-FDH detection, respectively. The images are recorded and analyzed through Metamorph Software (Universal Imaging). Full-width half-maximum values are calculated using OriginPro 8 software.

## Supporting Information Available

Theoretical volumetric flow rate and residence time calculations, standard curve for light absorption vs NADH concentration, and images of custom-made PMMA flow cells (PDF)
