# Supplementary material for: Solid-Binding Peptide-Guided Spatially Directed Immobilization of Kinetically Matched Enzyme Cascades in Membrane Nanoreactors
Source: ACS Omega. 2021 Oct 4;6(41):27129–39. doi: 10.1021/acsomega.1c03774 (PMC8529655; doi:10.1021/acsomega.1c03774)
Supplement: Supplementary file 1 — ao1c03774_si_001.pdf [file ao1c03774_si_001.pdf]

Supporting Information

Solid-Binding Peptide Guided Spatially Directed Immobilization of Kinetically Matched Enzyme Cascades in Membrane Nanoreactors

Deniz T. Yucesoy[†,‡], Susrut Akkineni[†], Candan Tamerler[§], Bruce J. Hinds[*,†] and Mehmet Sarikaya[*,†,‡]

†Department of Materials Science and Engineering, University of Washington, Seattle, WA, 98195 USA

‡Department of Bioengineering, Izmir Institute of Technology, Urla, Izmir, 35430, Turkey

§Department of Mechanical Engineering, Institute for Bioengineering Research, University of Kansas, Lawrence, KS, 66045 USA

Supplementary Figures:

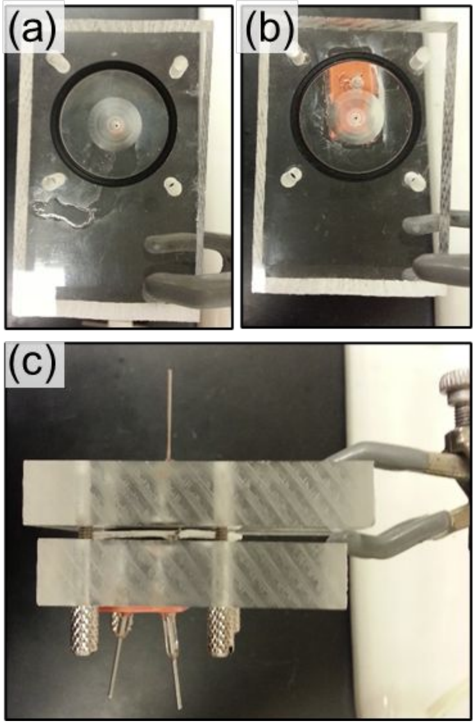

Figure S1: Representative images of a) top plate, b) bottom plate and c) after assembly of custom-made Plexiglas flow cells (Exposed membrane diameter of 1.8 cm).

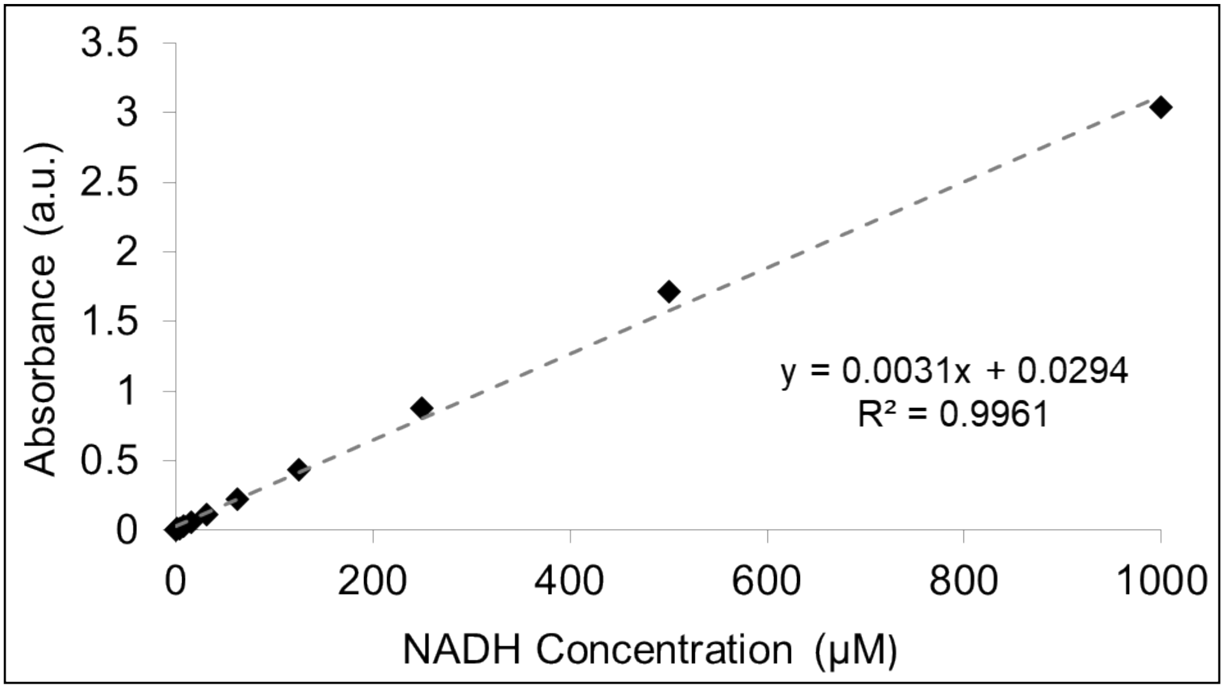

Figure S2: NADH calibration curve showing absorbance values of standard solutions at 340 nm wavelength with different NADH (0-10^{-3} M) concentrations.

Theoretical optimal volumetric flow rate and residence time calculations

The active gold coated surface area on the 20-200 nm pore size membrane was calculated to be 3.82 cm^2 using Equation S1 below, with pore density of 10^{11} cm^{-2}, AAO diameter of 1.82 cm, nominal pore diameter of 8.4 nm, and 20 nm Au sputter deposition depth. AuBP2-LDH active porous volume was 2.88 x 10^{-7} cm^3 (Volume of gold coated single pore x Pore density x Planar surface area of membrane). Experimentally measured mass and the corresponding number of AuBP2-LDH enzyme molecules (Molecular Weight $_{(tetramer)}$ = 1.52 x 10^5 g mol^{-1}) immobilized on the active electrode area was 2.2 x 10^{-7} g and ~ 8.6 x 10^{11} (tetrameric molecules), respectively. Using the Equation S2 and Equation S3 below, the theoretical flow rate and residence time was calculated to be 28.47 x 10^{-3} mL min^{-1} and 1.01 x 10^{-5} min, respectively.

Ni-NTA functionalization was performed to both top and bottom surfaces including the pore walls of the AAO membrane with 200-200 nm pore size. Using Equation S1 below, total active Ni-NTA area

was calculated as 787.96 cm^2 (pore density =10^9 cm^{-2}, AAO diameter = 1.82 cm, pore diameter = 160 nm and approximate Ni-NTA functionalized depth = 60 x 10^{-3} mm). Moreover, the enzyme activated volume was calculated to be 3.1 mm^3. Experimentally measured mass and the corresponding number of His_6-FDH enzyme molecules (Molecular Weight$_{(dimer)}$ = 8.57 x 10^4 g mol^{-1}) immobilized on the Ni-NTA active area was calculated to be 1.4 x 10^{-6} g and 9.9 x 10^{12} (dimeric molecules), respectively. Using the Equation S2 and Equation S3 below, theoretical flow rate and residence time of the FDH membrane was calculated to be 2.92 x 10^{-3} mL min^{-1} and 1.07 min, respectively.

$$A_{imm} = A_{face} + A_{pore\ wall} - A_{pore} \qquad \text{(S1)}$$

where A_{imm} (cm^2) is the active surface area for enzyme immobilization; A_{face} is area of functionalized face(s) of membrane, $A_{pore\ wall}$ is total functionalized surface area of inner pore walls and; A_{pore} is the area of pore throats on membrane face(s).

$$t_r = \frac{V_{total}}{N_{imm} \times TOF} \times C_{NADH} \qquad \text{(S2)}$$

where t_r is the residence time; V_{total} is enzyme activated volume, N_{imm} is total number of immobilized enzymes; TOF is the catalytic turnover rate of immobilized enzyme and; C_{NADH} is the initial cofactor concentration.[1]

$$Q = \frac{V_{total}}{t_r} \qquad \text{(S3)}$$

where Q is the volumetric flow rate; V_{total} is enzyme activated volume and; t_r is the residence time.

Fluorescent Labelling of Fusion Enzymes:

5-Carboxyfluorescein (5-FAM) was conjugated with *c*AuBP2-LDH[2] using 5-FAM protein labeling kit (Anaspec). Briefly, 5 x 10^{-5} M *c*AuBP2-LDH (in 10^{-2} M borate buffer, pH 7.4) was mixed with reaction buffer (component B) by vortexing. Then, 2 x 10^{-5} M 5-FAM solution (in Dimethyl sulfoxide) was added onto enzyme solution with 1:1 (v/v) ratio and incubated for 1 h at room temperature. Next, reaction was

quenched with 0.1 mL of glycine (0.1 M) solution for 1 h. The unreacted 5-FAM were removed by filtering the solution through 30 kDa cutoff ultrafiltration centrifugal unit (Amicon).

The Qdot-His-FDH[3] conjugates were prepared by conjugating the amine residues of His-FDH with carboxyl-coated Qdot 605 (ThermoScientific) through the EDC/sulfo-NHS chemistry. Briefly, Qdot stock solution was diluted in 10^{-2} M borate buffer (pH 7.4) to a final concentration of 1 x 10^{-7} M. Then, 0.5 mL of this solution was mixed with equal volume of His-FDH (5 x 10^{-5} M) solution. Conjugation reaction was started by adding freshly prepared 0.1 mL of EDC/NHS solution (1 mg/mL) and incubated for 2 h by continuous stirring at room temperature. The unreacted carboxyl groups were quenched with 0.1 mL of glycine (0.1 M) solution for 1 h. The unreacted Qdot were removed by purifying the solution through the Ni-NTA matrices. Finally, the unlabeled His_6-FDH was removed by filtering the solution through 100 kDa cutoff ultrafiltration centrifugal unit (Amicon). After labelling, both enzymes were exchanged into 5 x 10^{-2} M Tris, pH 7.5 buffer solution.

References

(1) Chen, Z.; Zhang, J.; Singh, S.; Peltier-Pain, P.; Thorson, J. S.; Hinds, B. J., Functionalized Anodic Aluminum Oxide Membrane–Electrode System for Enzyme Immobilization. ACS Nano **2014**, *8* (8), 8104-8112.

(2) Cetinel, S.; Caliskan, H. B.; Yucesoy, D. T.; Donatan, A. S.; Yuca, E.; Urgen, M.; Karaguler, N. G.; Tamerler, C., Addressable self-immobilization of lactate dehydrogenase across multiple length scales. Biotechnol. J. **2013**, *8* (2), 262-272

(3) Yucesoy, D. T.; Karaca, B. T.; Cetinel, S.; Caliskan, H. B.; Adali, E.; Gul-Karaguler, N.; Tamerler, C. Direct bioelectrocatalysis at the interfaces by genetically engineered dehydrogenase. Bioinspired, Biomimetic Nanobiomater. **2015**, *4* (1), 79-89.
